# Synergistic and stepwise treatment of resveratrol and catechol in *Haematococcus pluvialis* for the overproduction of biomass and astaxanthin

**DOI:** 10.1186/s13068-024-02527-z

**Published:** 2024-06-14

**Authors:** Jia-Fan Qiu, Yu-Cheng Yang, Ruo-Yu Li, Yu-Hu Jiao, Jin-Hua Mou, Wei-Dong Yang, Carol Sze Ki Lin, Hong-Ye Li, Xiang Wang

**Affiliations:** 1 Key Laboratory of Eutrophication and Red Tide Prevention of Guangdong Higher Education Institutes, College of Life Science and Technology, Jinan University, Guangzhou, 510632 China; 2 School of Energy and Environment, City University of Hong Kong, Hong Kong, China

**Keywords:** *Haematococcus pluvialis*, Astaxanthin, Resveratrol, Catechol, Stepwise cultivation strategies

## Abstract

To increase the production of biomass and astaxanthin from *Haematococcus pluvialis* to meet the high market demand for astaxanthin, this study recruited two typical and negligible phytohormones (namely resveratrol and catechol) for the stepwise treatments of *H. pluvialis*. It was found that the hybrid and sequential treatments of resveratrol (200 μmol) and catechol (100 μmol) had achieved the maximum astaxanthin content at 33.96 mg/L and 42.99 mg/L, respectively. Compared with the hybrid treatment, the physiological data of *H. pluvialis* using the sequential strategy revealed that the enhanced photosynthetic performance via the Calvin cycle by RuBisCO improved the biomass accumulation during the macrozooid stage; meanwhile, the excessive ROS production had occurred to enhance astaxanthin production with the help of NADPH overproduction during the hematocyst stage. Overall, this study provides improved knowledge of the impacts of phytohormones in improving biomass and astaxanthin of *H. pluvialis*, which shed valuable insights for advancing microalgae-based biorefinery.

**Supplementary Information:**

The online version contains supplementary material available at 10.1186/s13068-024-02527-z.

## Background

Astaxanthin, a naturally occurring fat-soluble ketone carotenoid, is highly esteemed for its potent antioxidant properties, which have been found to be extensively applied in medicine, cosmetics, dietary supplements, health products, and aquafeed [1–3]. It is estimated that the production value of astaxanthin can reach over USD 1 billion by 2025 [4]. Currently, commercial astaxanthins mainly contain two types, including synthetic astaxanthin and natural astaxanthin. It is reported that the antioxidant ability of natural astaxanthin is much higher than that of synthetic astaxanthin [5, 6], resulting in a growing market demand for large amounts of natural astaxanthin.

To date, several microbial species have been identified as capable of naturally synthesizing astaxanthin [2]. Among the unicellular green microalga *Haematococcus pluvialis* is identified as the most promising strain for the production of astaxanthin with a very high content (nearly 2–4% of dry cell weight [DCW]) of astaxanthin during the haematocyst stage, which has been achieved industrial-scale application [7, 8]. Nonetheless, relying solely on conventional cultivation of *H. pluvialis* is not a viable solution to address the market shortage of natural astaxanthin. Thus, it is urgent to develop novel strategies to enhance astaxanthin yield to meet the burgeoning global demand for natural astaxanthin.

Numerous efforts have been made to enhance the production of astaxanthin in *H. pluvialis*, including mutagenesis, abiotic stress, and exogenous chemical supplementation [9, 10]. The overproduction of astaxanthin in *H. pluvialis* could be caused by exogenous ethanol induction through the intracellular jasmonate pathway [11]. Generally, these treatments of *H. pluvialis* mainly cause the generation of excessive reactive oxygen species (ROS), which primarily contribute to the morphological change (green motile cell to red aplanospore cell) of *H. pluvialis* from the macrozooid stage to the haematocyst stage, accompanying with the hyperaccumulation of astaxanthin [12]. Among these treatments, exogenous phytohormone supplementation can be considered as the optimal solution to enhance the production yield of astaxanthin up to commercially viable levels [13, 14].

Phytohormones serve as regulators and signal messengers that control various cellular processes, including growth, reproduction, metabolism, and stress response. Given the accessibility and high efficiency of combining phytohormones to promote algal metabolite production, many researchers have adopted this approach [15, 16]. For instance, the treatment of typical phytohormone strigolactone analogue *rac*-GR24 in *H. pluvialis* led to the astaxanthin accumulation of up to 5.32% of DCW [17]. Similarly, it was found that fulvic acid improved the accumulation of astaxanthin and lipid contents in *H. pluvialis* under abiotic stress conditions [18].

Resveratrol (Res), a plant-derived compound, is produced in response to external stress. It functions as an antioxidant and signaling molecule, neutralizing the production of free radicals, activating antioxidant enzymes, and regulating the expression of functional genes during anti-stress procedures [19, 20]. The inclusion of Res stimulated the production of H_2_O_2_, thus activating the antioxidant enzyme system in peanut leaves [21]. Moreover, Res has been found to improve photosynthetic efficiency and mitigate abiotic stress in certain plant species [22]. Another phytohormone, catechol (Cat), has a similar function in regulating plant growth and acting as an antioxidant [23]. Additionally, in higher plants, Cat serves as an intermediate in the catabolism of salicylic acid, which confers tolerance to abiotic stress [24]. Since these phytohormones could regulate the intracellular oxidative system, which was responsible for the production of astaxanthin in *H. pluvialis*, therefore, it is possible to recruit these phytohormones for the treatment of microalgae. Thus far, it remains unclear the synergistic effects and possible mechanisms of *H. pluvialis* when treated with the joint treatments of Res and Cat, especially in the performance of biomass and astaxanthin.

This study first investigated the effects of Res and Cat at different concentrations and treatment time points for the production of biomass and astaxanthin in *H. pluvialis*, respectively. Subsequently, different strategies for the joint treatment of Res and Cat were implemented to evaluate the accumulation of biomass and astaxanthin. Physiological and biochemical responses were further determined to propose possible molecular mechanisms of joint treatment of Res and Cat for the production of biomass and astaxanthin. Overall, the study provides novel insights into the joint treatment of phytohormones for biomass and astaxanthin biosynthesis, offering a promising and suitable method for future astaxanthin biorefinery.

## Results and discussion

### Effects of Res and Cat on biomass and astaxanthin accumulation of *H. pluvialis*

Phytohormones have been widely recognized for their positive effects on the production of biomass and valuable products in microalgae; however, when selecting appropriate phytohormones for the treatment of microalgae, it is essential to consider the cost and availability of phytohormones [13, 17, 25]. Res and Cat, two commonly used phytohormones, were recruited in this study due to their reasonable cost and manure commercialization. A range concentration (100, 200, and 400 μmol) of Res and Cat was conducted to evaluate the effect of astaxanthin accumulation in *H. pluvialis*. As expected, the treatment of Res or Cat at day 0 stimulated astaxanthin biosynthesis, with 200 μmol of Res and 100 μmol of Cat resulting in the maximum astaxanthin content at 5.14% and 5.13% of the dry cell weight (DCW), respectively (Additional file 1: Fig. S1). Consequently, 200 μmol of Res and 100 μmol of Cat were chosen to investigate the impact of treatment time point on both biomass and astaxanthin accumulation.

Accordingly, *H. pluvialis* was treated with Res or Cat at three different treatment time points: day 0 (at the beginning of the macrozooid stage), day 6 (at the logarithmic period of the macrozooid stage), and day 12 (at the beginning of the hematocyst stage). Interestingly, the addition of 200 μmol of Res on day 0 led to the maximum biomass accumulation at day 12 (566.2 ± 41.8 mg/L) and day 16 (563.9 ± 29.7 mg/L), as shown in Table 1. This finding is consistent with previous studies on *Chlorella*, which demonstrated that the supplementation of the phytohormone indole-3-acetic acid promotes microalgal growth [26, 27]. Conversely, the treatment of 100 μmol of Cat on day 12 resulted in the highest astaxanthin content (5.31 ± 0.44% of DCW) on day 16. Similarly, a prior study reported that treating *H. pluvialis* with a synthetic strigolactone analogue rac-GR24 on day 12 achieved the maximum astaxanthin content on day 16 [17]. These findings indicate that the treatment time point of phytohormone significantly influences the metabolism trend of microalgae, leading to different bioaccumulation directions during the whole cultivation period.

**Table 1:** The growth and astaxanthin effect of *H. pluvialis* treated by phytohormone with different treatment day

Group	Treatment day	Cell density (× 10^4^ cells/mL)	Specific growth rate during the macrozooid stage (μ)	DCW (mg/L) at day 12	DCW (mg/L) at day 16	Astaxanthin (mg/L) at day 16	Astaxanthin (% DCW) at day 16
Control	n/a	24.2 ± 1.8	0.265	417.6 ± 27.5	419.2 ± 20.9	10.1 ± 0.87	2.42 ± 0.19
Resveratrol (200 μmol)	0	35.4 ± 0.9	0.297	566.2 ± 41.8	563.9 ± 29.7	29.3 ± 1.55	5.19 ± 0.28
Resveratrol (200 μmol)	6	29.7 ± 1.5	0.282	489.4 ± 32.1	492.1 ± 36.2	24.9 ± 1.94	5.07 ± 0.25
Resveratrol (200 μmol)	12	24.9 ± 1.9	0.268	424.1 ± 25.9	424.5 ± 32.4	20.7 ± 2.02	4.89 ± 0.31
Catechol (100 μmol)	0	23.6 ± 1.3	0.263	409.8 ± 35.6	410.1 ± 30.5	20.8 ± 2.16	5.08 ± 0.42
Catechol (100 μmol)	6	24.4 ± 0.7	0.266	418.3 ± 28.8	416.9 ± 26.6	21.5 ± 1.62	5.15 ± 0.36
Catechol (100 μmol)	12	24.8 ± 2.1	0.267	422.6 ± 33.4	423.8 ± 28.5	22.5 ± 2.09	5.31 ± 0.44

*n/a* not applicable

### Different stepwise cultivation strategies with Res and Cat caused different accumulation trends of biomass and astaxanthin

**Fig. 1:**
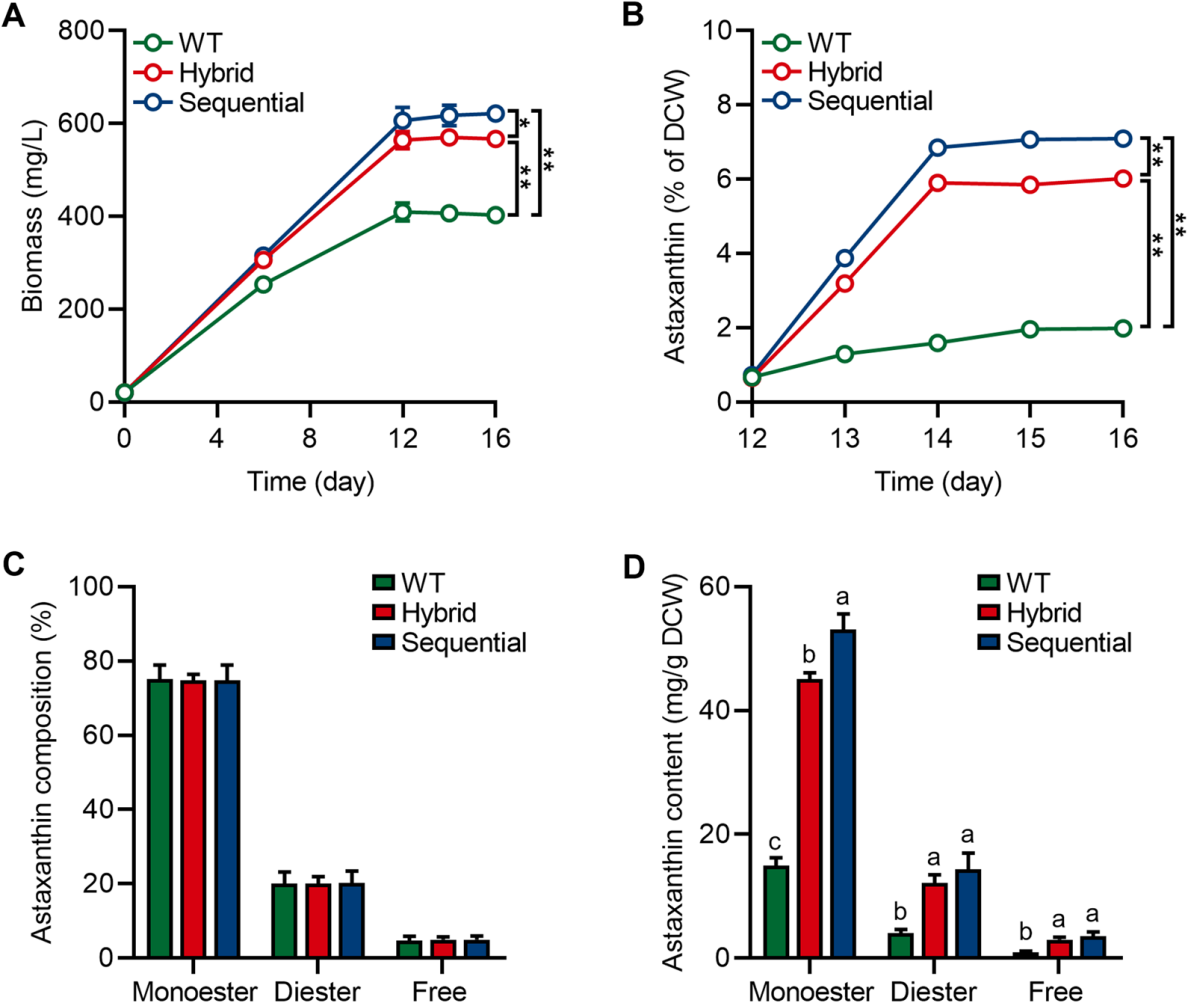
Biomass and astaxanthin profile of *H. pluvialis* treated with 200 μmol of Res and 100 μmol of Cat in hybrid and sequential feeding strategies. **A** Biomass content; **B** Astaxanthin content; **C** The relative ratio of astaxanthin compositions; **D** The content of astaxanthin compositions. Error bars in the figures represent the standard deviation of measurements from at least three samples. Significant differences (*p* < 0.05 marks * and *p* < 0.01 marks **) are indicated between groups. Different lowercase letters on the column bars indicate significant differences with *p* < 0.05

It is important to note that the cultivation strategies (namely addition modes of phytohormones) are crucial for the effective and simultaneous accumulation of biomass and valuable products in microalgae [28, 29]. On the other hand, the performance of Res or Cat in *H. pluvialis*, as influenced by the treatment day, has been demonstrated, which provided the positive effects of phytohormones for the accumulation of biomass and astaxanthin. Therefore, in this study, two stepwise cultivation strategies with 200 μmol of Res and 100 μmol of Cat, namely hybrid and sequential, were employed to assess their synergistic effects on growth enhancement and astaxanthin production. As shown in Fig. 1A, the highest biomass accumulation achieved at 564.1 mg/L and 606.3 mg/L in *H. pluvialis* was observed on day 12 under hybrid and sequential strategies, respectively, indicating a higher biomass accumulation effect using the sequential addition approach [28]. Furthermore, the stepwise cultivation strategies did not affect the biomass change during the hematocyst stage, consistent with the findings shown in Table 1. While the biomass attained in this study may seem slightly lower for industrial purposes, it is influenced by strain characteristics, inoculum concentration, and lab cultivation conditions. Therefore, in the future, it is essential to refine cultivation conditions and select optimal *H. pluvialis* species for high-density cultivation to bridge the gap between lab-scale investigation and large-scale production. Notably, the astaxanthin content in *H. pluvialis* treated with both hybrid and sequential strategies was significantly increased during the hematocyst stage with the highest content at 6.02% (33.96 mg/L) and 7.09% (42.99 mg/L) of DCW, respectively (Fig. 1B). The experimental results presented in this study illustrate the successful induction of astaxanthin biosynthesis in *H. pluvialis* using the stepwise cultivation strategies with Res and Cat.

It is worth mentioning that astaxanthin from *H. pluvialis* exists in three forms: monoester (approximately 75%), diester (approximately 20%), and free (approximately 5%) forms [30]. Furthermore, previous studies have shown that the bioavailability of the monoester form of astaxanthin is higher compared to the other forms [3, 31]. Thus, the astaxanthin profile of *H. pluvialis* was further investigated on day 16 after stepwise cultivation strategies with Res and Cat. The ratio of astaxanthin composition remained unchanged upon stepwise cultivation strategies with Res and Cat (Fig. 1C). Interestingly, due to the accumulation of astaxanthin content, the absolute content of each astaxanthin form increased in *H. pluvialis* (Fig. 1D). Consequently, the stepwise cultivation strategies of phytohormone treatment did not influence the ratio of each astaxanthin ester form. Moreover, on the other hand, the monoester and diester remained dominant, comprising approximately 95% of total astaxanthin content, regardless of the stepwise cultivation strategies with Res and Cat, which aligns with their higher abundance in *H. pluvialis* [30].

### Different stepwise cultivation strategies with Res and Cat improved photosynthesis and intracellular metabolites of *H. pluvialis*

**Fig. 2:**
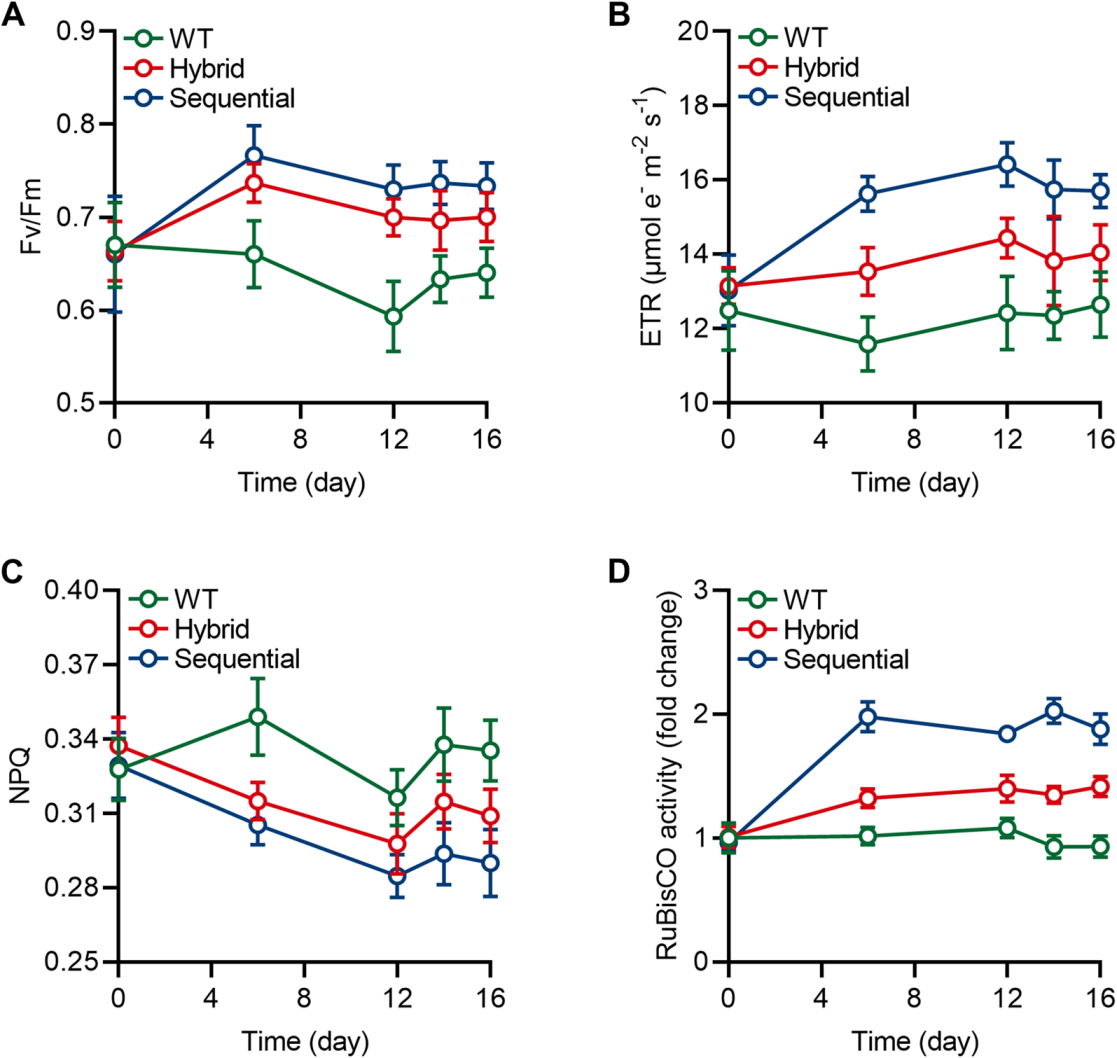
Photosynthetic profile of *H. pluvialis* treated with 200 μmol of Res and 100 μmol of Cat in hybrid and sequential feeding strategies. **A** The value of maximum photochemical efficiency of photosystem II (Fv/Fm); **B** The value of electron transport rate (ETR); **C** The value of non-photochemical quenching (NPQ); **D** The relative enzymatic activity of ribulose-1,5-bisphosphate carboxylase-oxygenase (RuBisCO). Error bars in the figures represent the standard deviation of measurements from at least three samples

To investigate the effects of stepwise cultivation strategies with Res and Cat on the physiological responses of *H. pluvialis*, the photosynthetic performance was assessed. The results, as shown in Fig. 2A–C, clearly demonstrated a remarkable change in photosynthetic parameters including Fv/Fm, ETR, and NPQ in *H. pluvialis* using different stepwise cultivation strategies with Res and Cat. These findings demonstrated that phytohormones improve microalgal photosynthetic efficiency, which is consistent with previous studies [32, 33]. Additionally, the photosynthetic parameters of *H. pluvialis* cultures using the sequential strategy exhibited significant improvements compared to those using the hybrid strategy (Fig. 2A–C). This suggests that external treatment of phytohormone has a beneficial impact on microalgal photosynthesis, thereby promoting the growth of *H. pluvialis* [15]. This observation aligns with a previous report that microalgal growth can be enhanced through a positive response of photosynthesis under the sequential cultivation strategy [28]. Furthermore, the Calvin–Benson–Bassham cycle, regulated by the enzyme ribulose-1,5-bisphosphate carboxylase-oxygenase (RuBisCO), has been implicated in microalgal photosynthesis [34]. As anticipated, the enzymatic activity of RuBisCO was found to be higher in *H. pluvialis* cultivated using stepwise cultivation strategies with Res and Cat compared to the control group (Fig. 2D). Moreover, the sequential strategy maintained the highest enzymatic activity throughout the entire treatment period, consistent with the results obtained for photosynthetic parameters and biomass. It can be concluded that stepwise cultivation strategies with Res and Cat in *H. pluvialis* enhance photosynthetic performance through the regulation of the Calvin–Benson–Bassham cycle by RuBisCO, resulting in the improvement of biomass during the macrozooid stage.

**Fig. 3:**
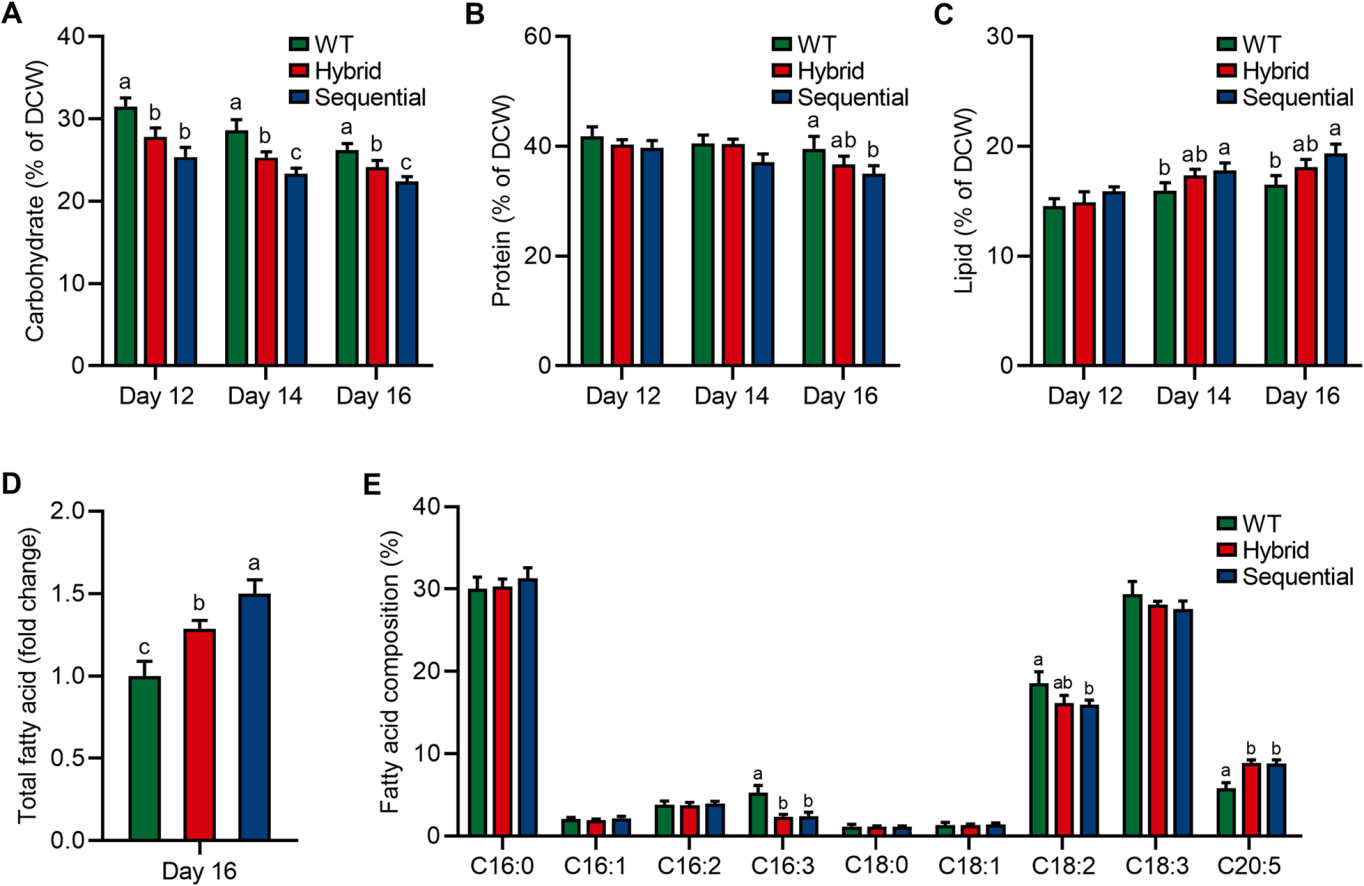
Primary metabolite profile of *H. pluvialis* treated with 200 μmol of Res and 100 μmol of Cat in hybrid and sequential feeding strategies. **A** Carbohydrate content; **B** Protein content; **C** Lipid content; **D** The relative content of total fatty acid; **E** The relative ratio of fatty acid composition. Error bars in the figures represent the standard deviation of measurements from at least three samples. Different lowercase letters on the column bars indicate significant differences with *p* < 0.05

The lipid-soluble nature of astaxanthin generally causes the concomitant accumulation of lipids during the hematocyst stage of *H. pluvialis*, especially when astaxanthin is overproduced [35]. This is due to the redirection of carbon precursors towards noncarbohydrate biosynthesis, which facilitates the formation of lipid-rich droplets necessary for astaxanthin assembly [17, 36]. In this context, the primary metabolites during the hematocyst stage were evaluated under stepwise cultivation strategies with Res and Cat. As shown in Fig. 3A, the overall trend of carbohydrate content exhibited a decreasing trend with cultivation time. Furthermore, the carbohydrate content of microalgae treated with Res and Cat under the sequential strategy was decreased from 25.37% to 22.39% of DCW. It is worth noting that the protein content of *H. pluvialis* with the hybrid strategy or without treatment remained relatively stable (36.71–41.89% of DCW) during the hematocyst stage (Fig. 3B). However, under the sequential strategy, protein content notably decreased (34.98% of DCW) compared to other treatments. Additionally, Fig. 3C demonstrated a gradual increase of lipid content in *H. pluvialis* during the hematocyst stage. As expected, the decreased carbohydrate and protein contents contributed to the accumulation of lipids in microalgae treated with Res and Cat using the sequential strategy, indicating a diversion of carbon flux from carbohydrate and protein biosynthesis towards lipogenesis (Fig. 3C). These findings align with previous studies that have shown the influence of phytohormones on carbon precursors, redirecting them towards noncarbohydrate biosynthesis [17, 37].

Alongside the accumulation of lipids during the production of astaxanthin at the hematocyst stage of *H. pluvialis*, there is a simultaneous process of fatty acid accumulation and esterification. This serves as a means for further astaxanthin esterification and storage [38, 39]. Consistent with the increased lipid content on day 16, the total fatty acid content increased by 1.29-fold and 1.50-fold under hybrid and sequential strategies compared to the control (Fig. 3D). Notably, the fatty acid profiles, such as C16:3, C18:2, and C20:5, were dominant in *H. pluvialis*, with significant changes in proportions under hybrid and sequential strategies (Fig. 3E).

### Different stepwise cultivation strategies with Res and Cat activated intracellular hormone metabolism and oxidative responses of *H. pluvialis*

A number of previous studies have indicated the effectiveness of exogenous phytohormone treatment in improving the metabolism of intracellular hormones in microalgae [17, 40]. Therefore, the levels of intracellular hormones, such as strigolactone (SL) and abscisic acid (ABA), in *H. pluvialis* were determined to evaluate the effects of stepwise cultivation strategies with Res and Cat. The SL content was significantly higher under the treatment of Res and Cat compared to the control (Additional file 1: Fig. S2A). In addition, *H. pluvialis* under the sequential strategy exhibited the highest SL content, aligning with the changes observed in astaxanthin levels. It is reported that SL derived from carotenoids could act as a typical phytohormone to influence biomass growth and regulate astaxanthin production [17, 41]. Thus, it is understandable that the synergistic treatment of Res and Cat in *H. pluvialis* stimulates SL production, promoting growth and astaxanthin production. On the other hand, SL generally interacts with ABA to regulate intracellular metabolism in plant [42]. As expected, the ABA levels in *H. pluvialis* were higher under stepwise cultivation strategies compared to the control (Additional file 1: Fig. S2B), confirming the collaborative accumulation between SL and ABA. Previous research has reported that increased intracellular ABA mainly promotes the growth of *H. pluvialis* [41], consistent with our findings which demonstrate that the elevated SL and ABA mainly contribute to the biomass accumulation of *H. pluvialis*.

To further validate the biosorption capability of exogenous phytohormones by *H. pluvialis*, the endogenous content of Res and Cat was determined throughout the entire cultivation period. The intracellular content of Res displayed a similar trend under the hybrid and sequential strategies (Additional file 1: Fig. S3A). Specifically, the sequential strategy resulted in slightly higher Res content in *H. pluvialis* compared to the hybrid strategy. Interestingly, while 100 μmol of Cat was added on day 0, the content of Cat gradually increased under the hybrid strategy (Additional file 1: Fig. S3B). In contrast, the sequential strategy led to a sharp increase in Cat content, reaching the highest level in microalgae cells. These data indicate a plausible antagonism between Res and Cat during the macrozooid stage of *H. pluvialis*, resulting in decreased intracellular Res levels and delayed biosorption of Cat, which finally led to the different physiological responses between the sequential and hybrid strategies.

**Fig. 4:**
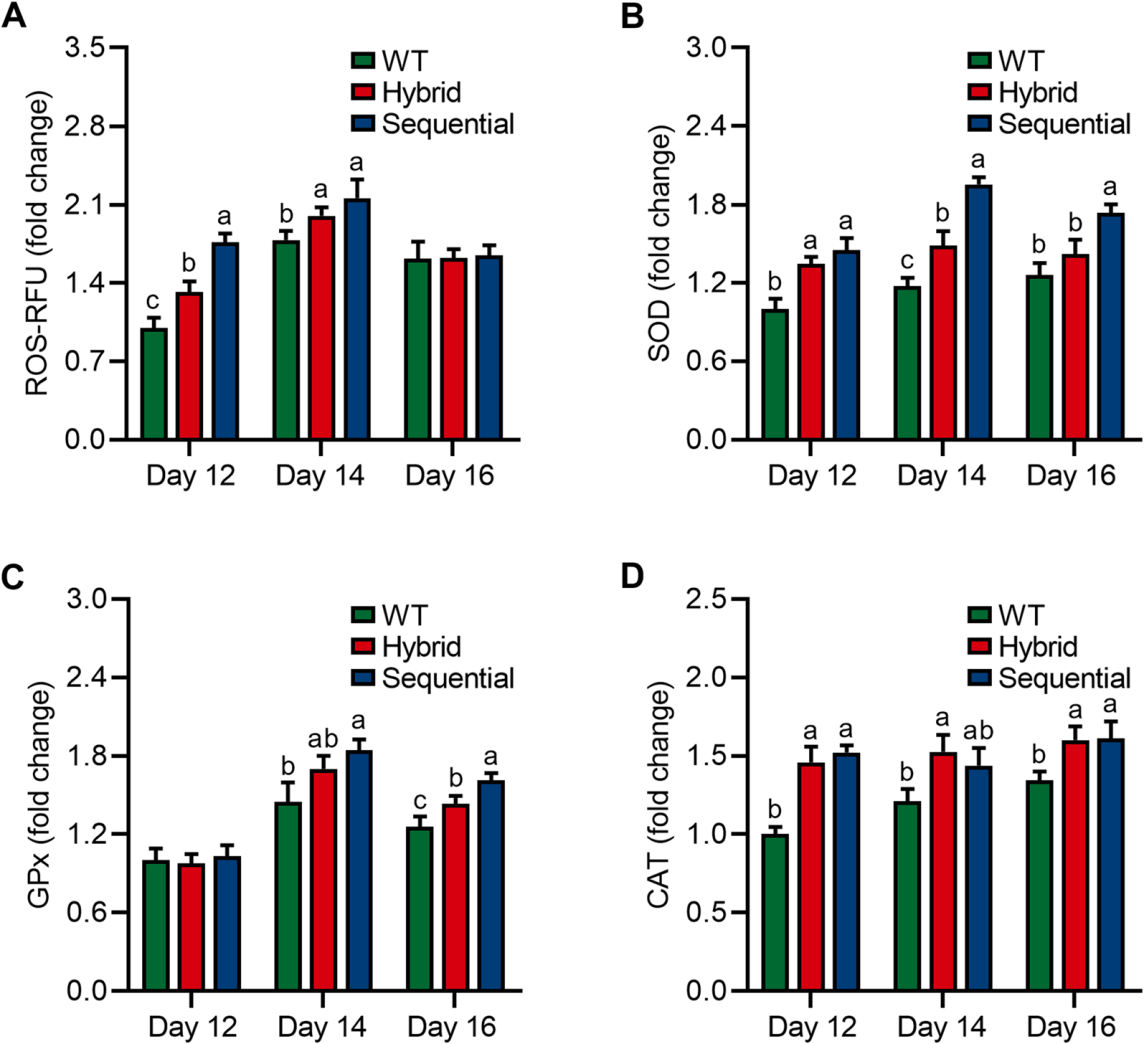
Oxidative profile of *H. pluvialis* treated with 200 μmol of Res and 100 μmol of Cat in hybrid and sequential feeding strategies. **A** Relative reactive oxygen species (ROS) content; **B** The relative enzymatic activity of superoxide dismutase (SOD); **C** The relative enzymatic activity of glutathione peroxidase (GPx); **D** The relative enzymatic activity of catalase (CAT). Error bars in the figures represent the standard deviation of measurements from at least three samples. Different lowercase letters on the column bars indicate significant differences with *p* < 0.05

Oxidative stress is known to play a crucial role in regulating the production of astaxanthin at the hematocyst stage in *H. pluvialis* [43, 44]. It is hypothesized that the excessive generation of reactive oxygen species (ROS) leads to oxidative stress, which in turn promotes the accumulation of astaxanthin as a defense mechanism against oxidative damage. To test this hypothesis, the ROS levels of *H. pluvialis* at the hematocyst stage were measured. The results showed that the stepwise cultivation strategies with Res and Cat significantly induced ROS generation at the beginning of the hematocyst stage, which might contribute to an increase in the production of astaxanthin (Fig. 4A). Additionally, on day 12, the ROS levels in *H. pluvialis* treated with the sequential strategy achieved the maximum value (2.16-fold), confirming that excessive ROS signaling stimulates astaxanthin production when treated with phytohormone inducers [12, 45]. The activities of key antioxidative enzymes, including SOD, GPx, and CAT, can be affected by ROS production and antioxidative products within microalgae cells [46]. As expected, in the presence of Res and Cat, the activities of SOD, GPx, and CAT were significantly higher during the hematocyst stage (Fig. 4B-D), indicating that the treatment of Res and Cat improves the enzymatic activities of the antioxidative system [12]. On the other hand, the improvement of enzymatic activities of the antioxidative system might also contribute to the consumption of astaxanthin to alleviate oxidative stress during the hematocyst stage, leading to the accumulation of astaxanthin [17, 36]. These results demonstrate that the addition of Res and Cat stimulates excessive ROS production to enhance astaxanthin production, with the latter contributing to improved ROS-scavenging capacities by increasing the enzymatic antioxidative system.

A previous study has shown that adenosine triphosphate (ATP), a crucial energy molecule, may alleviate cellular oxidative damage resulting from excessive ROS generation [47]. This excessive production of ATP, induced by supplying Res and Cat to *H. pluvialis* (Additional file 1: Fig. S4A), could contribute to the alleviation of oxidative damage, with the aid of antioxidant enzymes. On the other hand, excessive ATP may also be involved in other metabolic processes, such as biomass and astaxanthin accumulation [17, 36], which is consistent with our findings. NADPH serves as a key reducing agent involved in the biosynthesis of intracellular valuable products and also aids in the alleviation of oxidative damage [48]. Both feeding strategies exhibited an upward trend in NADPH levels, with the sequential feeding strategy demonstrating higher NADPH levels compared to the hybrid strategy involving Res and Cat (Additional file 1: Fig. S4B). It is thus reasonable to speculate that the overproduced NADPH in *H. pluvialis* is responsible for astaxanthin production and ROS elimination. Overall, stepwise cultivation strategies with Res and Cat in *H. pluvialis* caused the enhancement of ATP and NADPH, thereby leading to the accumulation of biomass and astaxanthin.

### Plausible molecular mechanisms of *H. pluvialis* with different cultivation strategies of phytohormone treatment

**Fig. 5:**
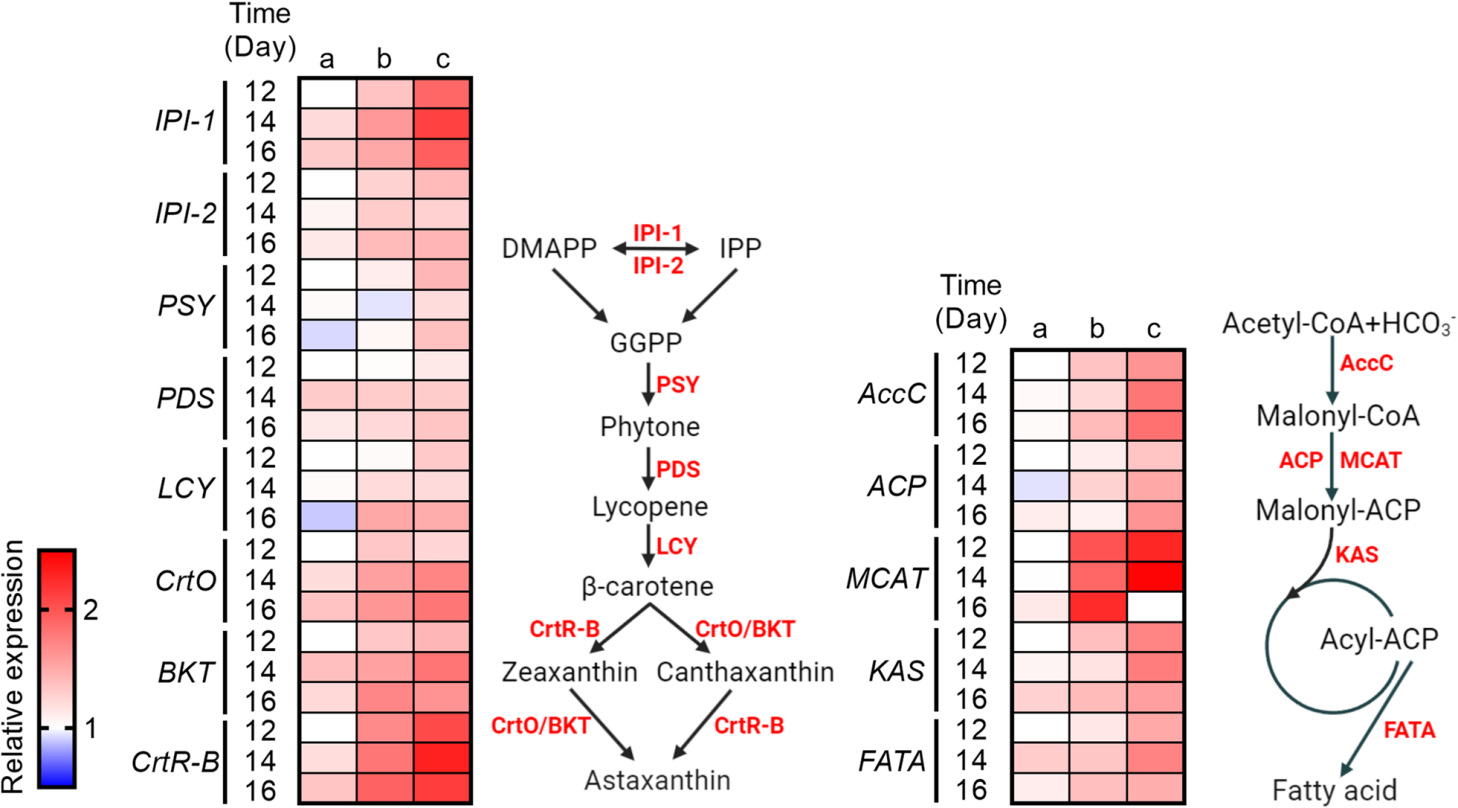
Relative abundances of related metabolisms in *H. pluvialis* treated with 200 μmol of Res and 100 μmol of Cat in hybrid and sequential feeding strategies. a represents the group of *H. pluvialis* without treatment, b represents the group of *H. pluvialis* with Res and Cat treatments in the hybrid strategy, c represents the group of *H. pluvialis* with Res and Cat treatments in the sequential strategy. All data were performed on at least biological triplicates

**Fig. 6:**
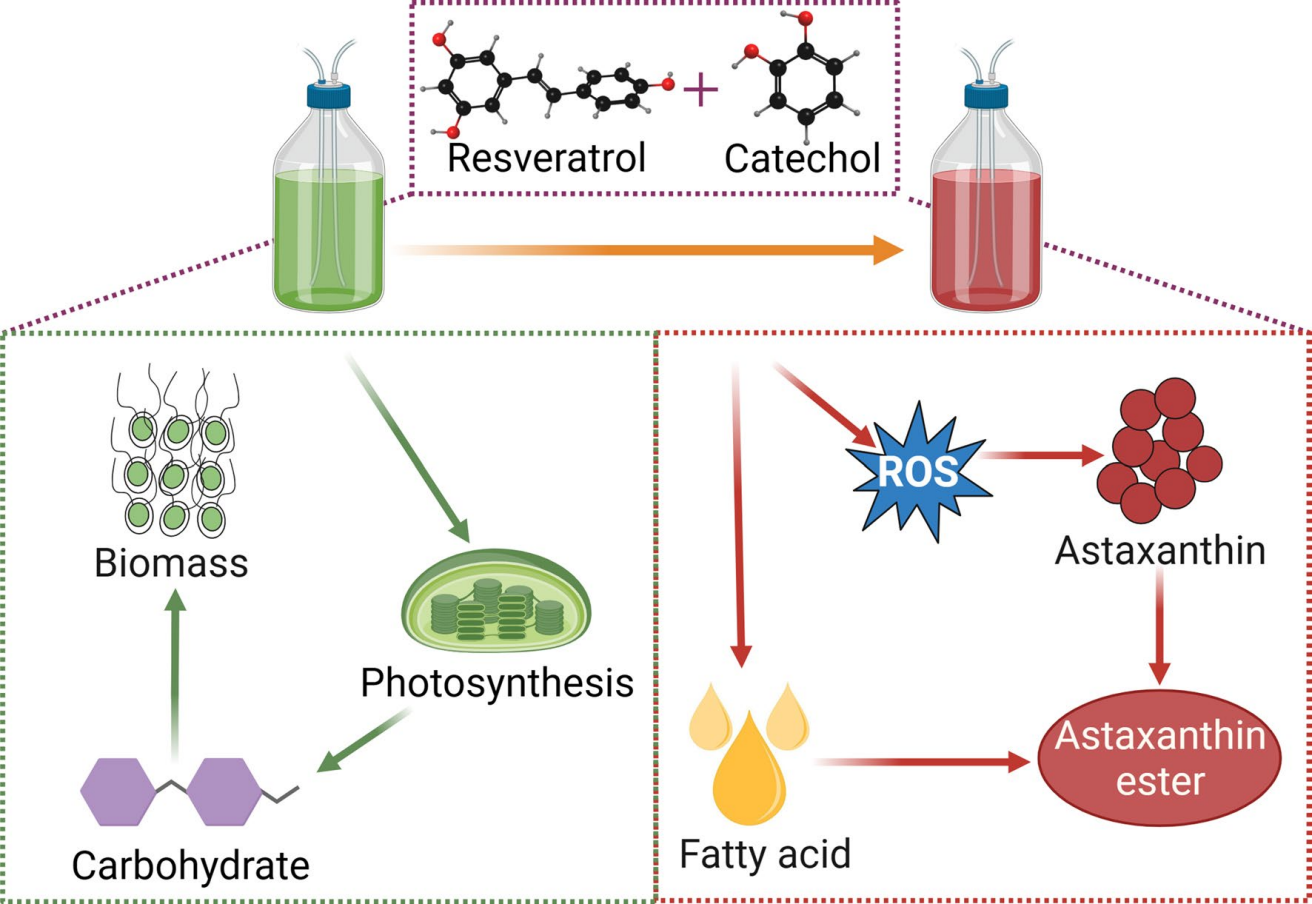
Proposed mechanism of the treatment of Res and Cat in the accumulation of biomass and astaxanthin in *H. pluvialis*

Further investigation is necessary to obtain a detailed understanding of the molecular interactions involved in astaxanthin accumulation [17]. To gain insight into the mechanistic roles of stepwise cultivation strategies with Res and Cat in the astaxanthin accumulation of *H. pluvialis* during the hematocyst stage, the expression abundances of key lipogenic and carotenogenic genes were determined. Firstly, it was observed that the expression pattern of several genes involved in the astaxanthin biosynthetic pathway was upregulated when treated with Res and Cat (Fig. 5). This suggests that stepwise cultivation strategies with Res and Cat activate the overexpression of carotenogenic genes, thereby leading to the accumulation of astaxanthin. Previous studies have also reported similar findings, where exogenous phytohormone treatment stimulates the transcript abundance of genes at the molecular level to achieve overproduction of valuable products in microalgae [49, 50]. Additionally, the mRNA level of fatty acid-related genes was significantly upregulated by stepwise cultivation strategies with Res and Cat, indicating that the induction of Res and Cat in *H. pluvialis* can trigger the transcript expression of fatty acid biosynthesis (Fig. 5), thus enhancing fatty acid production. Together, these results provide a plausible molecular mechanism for the perturbation of transcription in astaxanthin and fatty acid biosynthesis mediated by stepwise cultivation strategies with Res and Cat (Fig. 6).

### A preliminary evaluation hinted the profitable and feasible of stepwise cultivation strategies with Res and Cat

The alignment of mass balance performance in microalgae-based valuable product accumulation with the principles of commercial products is well established and serves as a validation of feasibility for future industrial applications [51, 52]. Thus, the evaluation of mass balance using stepwise cultivation strategies in microalgae-based astaxanthin production is necessary. In this study, a process flowchart delineating the input and output materials was generated for the sequential strategy of the production of astaxanthin from *H. pluvialis* cultivation (Additional file 1: Fig. S5). The process was scaled to 1,000-L bioreactors for cultivating *H. pluvialis* using the sequential strategy with Res and Cat over a 16-day period based on the above-mentioned laboratory data, which could provide the preliminary evaluation for economic and environmental assessment in the future. Briefly, 45.6 g of Res and 11.1 g of Cat were utilized for microalgae cultivation based on the sequential manner, culminating in 600 g of the ultimate biomass with 42 g of astaxanthin content. This mass balance of microalgae-based astaxanthin production demonstrates that high quality of astaxanthin can be contributed from *H. pluvialis* using the sequential strategy with 200 μmol of Res and 100 μmol of Cat, implying the technically feasible astaxanthin production can be achieved by microalgae biorefinery. This feeding strategy using Res and Cat for astaxanthin production from *H. pluvialis* with higher biomass production should be further explored using the optimizations of stain selection, inoculum concentration and cultivation condition to provide insights for industrial application. Moreover, a systematic evaluation should be further executed to inspect the feasibility and sustainability of economic, environmental and social aspects in practical application.

## Conclusions

In this study, the optimal concentration and feeding time were successfully screened to achieve high-quality production of biomass and astaxanthin in *H. pluvialis*. Furthermore, different stepwise cultivation strategies (e.g., hybrid and sequential) were developed to enhance the production of astaxanthin, as well as the microalgae-based physiological and biochemical responses. The sequential strategy was observed to up-regulate the expression of pivotal genes associated with astaxanthin and fatty acid synthesis, consequently stimulating astaxanthin production. Additionally, a preliminary evaluation utilizing the sequential strategy at a scale of 1,000 L also showcased the feasibility of astaxanthin synthesis incorporating Res and Cat. In total, this study provides a new perspective on the enhanced biomass and astaxanthin production in *H. pluvialis* through stepwise cultivation strategies with Res and Cat.

## Materials and methods

### Strain and maintenance of microalgae

The freshwater and unicellular microalga *Haematococcus pluvialis* (No: CCMP-3127) was retrieved from the Provasoli-Guillard National Center for Marine Algae and Microbiota (East Boothbay, USA). The strain cultivated in the logarithmic growth phase at an initial density of 1 × 10^4^ cells/mL was subcultured into fresh Bold Basal Medium (BBM) without carbon dioxide supply, which was sterilized with a 0.22 μm pore-size filter membrane, with a total volume of 400 mL in Duran bottle. The cultivation condition was set up as follows: 25 ± 0.5 °C, 120 μmol·m^–2^·s^–1^ of light intensity at the macrozooid stage (day 0–12) and 400 μmol·m^–2^·s^–1^ of light intensity at the hematocyst stage (day 12–16), and 12/12 h of day/night cycle.

### Stepwise cultivation strategies of phytohormones on *H. pluvialis*

Two phytohormones, resveratrol (Res) and catechol (Cat), with purities of ≥ 99% (Macklin, China) were chosen for the treatment of *H. pluvialis* due to their regulatory and antioxidant properties. In the preliminary experiment, different concentrations of Res and Cat (100, 200, 400 μmol) were used on day 0 to evaluate the effects of *H. pluvialis* as a typically effective concentration range. Afterwards, the optimal concentration of Res and Cat were selected for the treatment of *H. pluvialis* at various time points to assess their effects on biomass and astaxanthin content. To evaluate the synergistic effect of two phytohormones in *H. pluvialis*, two stepwise cultivation strategies, named hybrid and sequential strategies, were conducted in subsequent experiments. Briefly, in the hybrid strategy, 200 μmol of Res and 100 μmol of Cat were added into the culture medium simultaneously on day 0 for the treatment of *H. pluvialis*. In the sequential strategy, 200 μmol of Res was added on day 0, and 100 μmol of Cat was added on day 12. All stepwise cultivation strategies with Res and Cat were conducted in triplicate, and microalgae samples were harvested at preset time points for further analysis.

### Determination of microalgal growth

The density of *H. pluvialis* was determined on a daily basis through direct counting with a Neubauer hemocytometer under an optical microscope. The specific growth rate (μ) of *H. pluvialis* was derived using Eq. 1:

1 $$\mu { }\left( {{\text{day}}^{ - 1} } \right) = \frac{{lnN - lnN_{0} }}{{t - t_{0} }},$$

where *N* and *N*_*0*_ represent the density of *H. pluvialis* at times *t* and *t*_*0*_, respectively, during the logarithmic growth phase.

For biomass assessment, 100 mL of microalgae was collected and resuspended in pre-weighed 1.5-mL Eppendorf tubes with distilled water. The resultant pellets were subjected to drying at 60 °C overnight for further gravimetric determination using an analytic balance.

### Measurement of microalgal photosynthesis

The photosynthetic parameters of *H. pluvialis*, such as the maximum photochemical efficiency of photosystem II (Fv/Fm), the electron transport rate (ETR), and the non-photochemical quenching (NPQ) of chlorophyll, were monitored using a PhytoPAM chlorophyll fluorometer (Heinz Walz GmbH, Germany) following standard instructions. Firstly, the instrument is set up according to the manufacturer’s instructions, and the sample is dark-adapted for 20–30 min to allow all PSII reaction centers to open. The PhytoPAM probe is then attached to the tube containing microalgae, ensuring good contact, and the measurement sequence is initiated. For Fv/Fm calculation, the formula (Fm—Fo) / Fm is used, where Fm represents the maximum fluorescence yield and Fo the minimum fluorescence yield in the dark-adapted state. Following Fv/Fm measurement, the sample is exposed to actinic light of known intensity to measure light-adapted fluorescence (F’) using PhytoPAM. ETR is then calculated using a formula incorporating ΔF’ (change in fluorescence between light-adapted and dark-adapted states), F’m (maximum fluorescence yield in light-adapted state), PAR (photosynthetically active radiation), and conversion factors. NPQ is determined by measuring light -adapted fluorescence (F’) and maximum fluorescence yield during a saturating light pulse (Fm’), then applying the formula (Fm’—F’m)/F’m.

To assess the efficiency of photosynthesis, the enzymatic activity of ribulose-1,5-bisphosphate carboxylase-oxygenase (RuBisCO), which is a key photosynthetic enzyme, was measured. In brief, 100 mL of microalgae was collected for grinding and subsequently transferred into a mixed solution composed of 20 mM magnesium chloride, 1 mM ethylenediaminetetraacetic acid, 10 mM sodium bicarbonate, 50 mM 4-(2-hydroxyethyl)-1-piperazine ethanesulfonic acid, and 10 mM β-mercaptoethanol to isolate intracellular crude enzymes of *H. pluvialis*. The RuBisCO activity of *H. pluvialis* was determined at an absorbance wavelength of 340 nm using UV–Vis spectrophotometry, following the previously established protocol [53].

### Detection of microalgal primary metabolites

The total carbohydrate content of *H. pluvialis* was determined using the conventional phenol–sulfuric acid method with slight modifications. Specifically, 100 mL of microalgae was collected and then transferred into a mixed solution consisting of 1 mL of distilled water and 1 mL of 5% phenol solution (*w*/*v*), followed by the dropwise addition of 5 mL of 98% sulfuric acid along the tube wall. The mixture was incubated at 25 °C for 20 min and subsequently analyzed at an absorbance wavelength of 483 nm using UV–Vis spectrophotometry. The concentration of intracellular carbohydrate in *H. pluvialis* was normalized with the standard curve of a range of glucose concentrations.

The total protein of *H. pluvialis* was isolated using protein lysis buffer (Beyotime, China) supplied with the protease inhibitor phenylmethylsulfonyl fluoride (Beyotime, China), as instructed by the manufacturer. The microalgal protein content was quantified using the BCA Protein Assay kit (Beyotime, China) following the standard protocols provided by the manufacturer.

The total lipid content of *H. pluvialis* was determined gravimetrically using the conventional methanol–chloroform method with slight modifications. In brief, 200 mL of microalgae was harvested for further lyophilization. The lyophilized precipitation was ground under liquid nitrogen and transferred into a falcon tube containing a mixture solution of methanol, chloroform, and water at a volume ratio of 2:1:0.8. The solution was then subjected to ultrasonication at 200 W for 15 min. Subsequently, 2 mL of mixture solution containing chloroform and water at a volume ratio of 1:1 was added to the falcon tubes, followed by the gentle vortex. The lower phase of the solution, obtained after centrifugation at 2,000 rpm for 5 min, was collected into a pre-weighed Eppendorf tube and dried using a nitrogen stream for gravimetric determination.

The fatty acids of *H. pluvialis* were then transesterified into fatty acid methyl esters following previous protocols and analyzed using gas chromatography–mass spectrophotometry equipped with a NIST 147 spectrum library for fatty acid quantification based on the normalization of peak area for each fatty acid [54].

### Analysis of microalgal astaxanthin

A total of 100 mL of microalgae was harvested and then transferred into a 10-mL Falcon tube to which 600 μL of a 5% potassium hydroxide solution was added. The resulting mixture was incubated in a dry bath incubator at 65 °C for 10 min and subsequently isolated by centrifugation at a speed of 6,000 rpm for 5 min. The isolated precipitate was then combined with 300 μL of dimethylsulfoxide and 300 μL of acetic acid for astaxanthin extraction. This extraction process was carried out in a dry bath incubator at 75 °C for 5 min. Three forms of astaxanthin, namely monoester, diester, and free form, were pretreated by saponification using a 21 mM solution of sodium hydroxide at room temperature for 3 h in darkness. The astaxanthin content present in the extracted samples was first passed through a 0.22-μm pore-size filter membrane and then analyzed using high-performance liquid chromatography (HPLC, Waters, UK) equipped with a reversed-phased C18 column (Waters, UK) as per the previous guideline [17]. The 10-μL samples underwent analysis using a photodiode array (PDA) detector (Waters, UK) at 460 nm. The eluents comprised two solutions: (A) dichloromethane, methanol, acetonitrile, and water in volume ratios (%) of 5:85:5.5:4.5, and (B) dichloromethane, methanol, acetonitrile, and water in volume ratios (%) of 25:28:42.5:4.5. The column was eluted at a flow rate of 1 mL/min, initially with 100% A for 8 min, transitioning to 100% B over 6 min, and maintained at 100% B for an additional 40 min. The quantification of astaxanthin from *H. pluvialis* was determined based on peak areas normalized from the reference standard.

### Determination of intracellular phytohormones

A total of 200 mL of microalgae was centrifuged at a speed of 3,000 × *g* and subsequently transferred into a clean glass tube. The resultant pellets were washed three times with distilled water to remove any residual culture medium and then ground using liquid nitrogen. Subsequently, 1 mL of pre-cold phosphate buffer (10 mM at pH = 7.4) was added into the pulverized microalgae for supernatant isolation by centrifugation at 12,000 rpm for 10 min at a temperature of 4 °C. The intracellular phytohormones, including strigolactone (SL) and abscisic acid (ABA), as well as Res and Cat of *H. pluvialis*, were identified by HPLC–MS following the standard protocols using the collected supernatant with filtration using a 0.22-μm pore-size filter membrane [55].

### Detection of microalgal antioxidant systems

To determine the levels of reactive oxygen species (ROS) in *H. pluvialis*, a cell-permeable fluorogenic probe, 2’,7’-dichlorodihydrofluorescein diacetate (DCFH-DA, Beyotime, China), was recruited using a microplate reader (Bio-Tek, USA) with an excitation wavelength of 488 nm and an emission wavelength of 500–600 nm. The enzymatic activities of intracellular superoxide dismutase (SOD), glutathione peroxidase (GPx), and catalase (CAT) were determined using commercially available kits (Beyotime, China) as per the provided instructions. The relative abundance of reduced nicotinamide adenine dinucleotide phosphate (NADPH) in *H. pluvialis* was assessed using the NADPH Determination Assay Kit (AAT Bioquest, USA) according to the manufacturer’s protocols. The adenosine triphosphate (ATP) content of *H. pluvialis* was measured using a Plant ATP enzyme-linked immunosorbent assay kit (Bangyi Biotech, China) according to the specifications provided by the manufacturer.

### Transcript abundance analyses of microalgal genes

The intracellular transcript abundance of genes, including *IPI-1* and *IPI-2* (which encode isoprenyl diphosphate isomerase), *PSY* (which encodes phytoene synthase), *PDS* (which encodes phytoene desaturase), *LCY* (which encodes lycopene cyclase), *CrtO* (which encodes β-carotene oxygenase), *BKT* (which encodes β-carotene ketolase), and *CrtR-B* (which encodes β-carotene hydroxylase) in astaxanthin synthesis, as well as *AccC* (which encodes biotin carboxylase), *ACP* (which encodes acyl carrier protein), *MCAT* (which encodes malonyl-CoA:acyl carrier protein transacylase), *KAS* (which encodes 3-ketoacyl-ACP synthase), and *FATA* (which encodes acyl-ACP thioesterase) in fatty acid synthesis were determined using real-time quantitative PCR (RT-qPCR). The Plant Total RNA Isolation kit (Sangon, China) was used for the extraction of the total RNA from *H. pluvialis* cultures. Subsequently, cDNA was transcribed from the total RNA using the HiScript II Q RT SuperMix for qPCR (Vazyme, China). RT-qPCR assay was conducted with diluted cDNA, 10 μM primers, 2 × AceQ qPCR SYBR Green Master Mix buffer, and distilled water to a final volume of 20 μL in 8-strip qPCR tubes. A CFX96 Real-Time PCR Detection System (Bio-rad, USA) was used for RT-qPCR assay with the standard manual. The data of RT-qPCR were normalized to the expression levels of 18 s rRNA, namely the reference gene, following the standard 2^–ΔΔCt^ method.

### Statistical analysis

To validate the normalcy of the data and the homogeneity of variance, the Shapiro–Wilk test and Levene’s test were performed, respectively. Differences among groups were analyzed using Kruskal–Wallis one-way analysis of variance (ANOVA), followed by Duncan’s multiple range test at a significance level of *p* < 0.05. Any significant differences were denoted by lowercase letters on the bars of the columns. Differences between the control group and the treatment group were assessed using an unpaired Welch’s t-test with statistical significance indicated as *p* < 0.05 (*) or *p* < 0.01 (**).

### Supplementary Information

Supplementary material 1: Fig. S1. Astaxanthin content of *H. pluvialis* treated with different concentrations of phytohormone, respectively. Fig. S2. The relative content of intracellular phytohormones in *H. pluvialis* cells treated with 200 μmol of Res and 100 μmol of Cat in hybrid and sequential feeding strategies. (A) The content of strigolactone; (B) The content of abscisic acid. Fig. S3. The content of exogenous phytohormones in *H. pluvialis* cells treated with 200 μmol of Res and 100 μmol of Cat in hybrid and sequential feeding strategies. (A) The content of Res; (B) The content of Cat. Fig. S4. The content of energy and reducing equivalent in *H. pluvialis* cells treated with 200 μmol of Res and 100 μmol of Cat in hybrid and sequential feeding strategies. (A) The content of ATP; (B) The content of NADPH. Fig. S5. Mass balance of *H. pluvialis* treated with 200 μmol of Res and 100 μmol of Cat in the sequential feeding strategy in a simulated 1,000-L scale based on the laboratory data from this study.

## Data Availability

All data supporting the conclusions of this article are included in this published article and its supplementary information files.
