# Supplementary material for: Synergistic and stepwise treatment of resveratrol and catechol in Haematococcus pluvialis for the overproduction of biomass and astaxanthin
Source: Biotechnol Biofuels Bioprod. 2024 Jun 14;17:80. doi: 10.1186/s13068-024-02527-z (PMC11177449; doi:10.1186/s13068-024-02527-z)
Supplement: Supplementary file 1 — Supplementary material 1: Fig. S1. Astaxanthin content of H. pluvialis treated with different concentrations of phytohormone, respectively. Fig. S2. The relative content of intracellular phytohormones in H. pluvialis cells treated with 200 μmol of Res and 100 μmol of Cat in hybrid and sequential feeding strategies. (A) The content of strigolactone; (B) The content of abscisic acid. Fig. S3. The content of exogenous phytohormones in H. pluvialis cells treated with 200 μmol of Res and 100 μmol of Cat in hybrid and sequential feeding strategies. (A) The content of Res; (B) The content of Cat. Fig. S4. The content of energy and reducing equivalent in H. pluvialis cells treated with 200 μmol of Res and 100 μmol of Cat in hybrid and sequential feeding strategies. (A) The content of ATP; (B) The content of NADPH. Fig. S5. Mass balance of H. pluvialis treated with 200 μmol of Res and 100 μmol of Cat in the sequential feeding strategy in a simulated 1,000-L scale based on the laboratory data from this study. [file 13068_2024_2527_MOESM1_ESM.docx]

**Synergistic and stepwise treatment of resveratrol and catechol in *Haematococcus pluvialis* for the overproduction of biomass and astaxanthin**

Jia-Fan Qiu^a,1^, Yu-Cheng Yang^a,1^, Ruo-Yu Li^a^, Yu-Hu Jiao^a^, Jin-Hua Mou^b^, Wei-Dong Yang^a^, Carol Sze Ki Lin^b^, Hong-Ye Li^a^, Xiang Wang^a,*^

^a^ Key Laboratory of Eutrophication and Red Tide Prevention of Guangdong Higher Education Institutes, College of Life Science and Technology, Jinan University, Guangzhou 510632, China

^b^ School of Energy and Environment, City University of Hong Kong, Hong Kong, China

*Corresponding author: Xiang Wang, Email: xiangwang@jnu.edu.cn.

^1^ These authors made equal contributions.


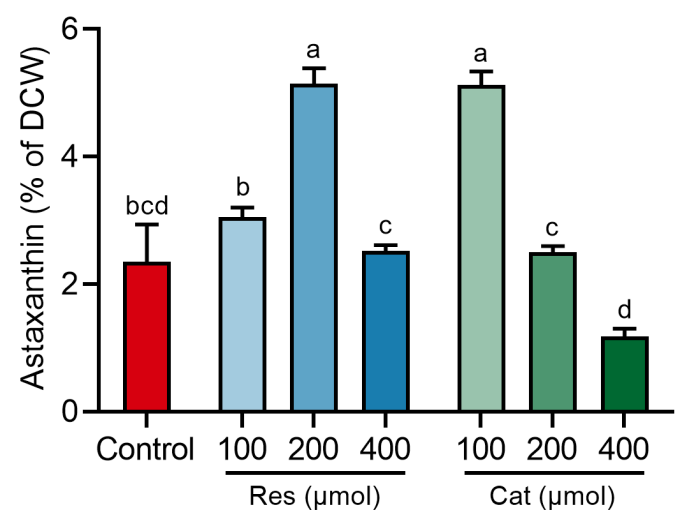


**Fig. S1.** Astaxanthin content of *H. pluvialis* treated with different concentrations of phytohormone, respectively. Error bars in the figures represent the standard deviation of measurements from at least three samples. Different lowercase letters on the column bars indicate significant differences with *p* < 0.05.


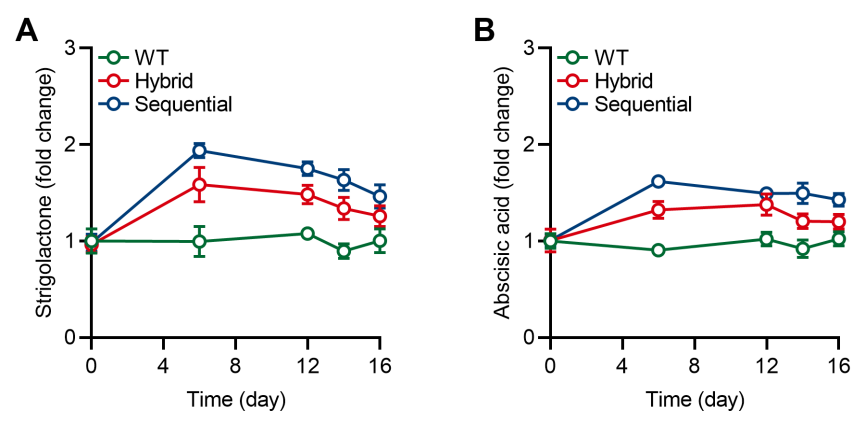


**Fig. S2.** The relative content of intracellular phytohormones in *H. pluvialis* cells treated with 200 μmol of Res and 100 μmol of Cat in hybrid and sequential feeding strategies. (A) The content of strigolactone (SL); (B) The content of abscisic acid (ABA). Error bars in the figures represent the standard deviation of measurements from at least three samples.


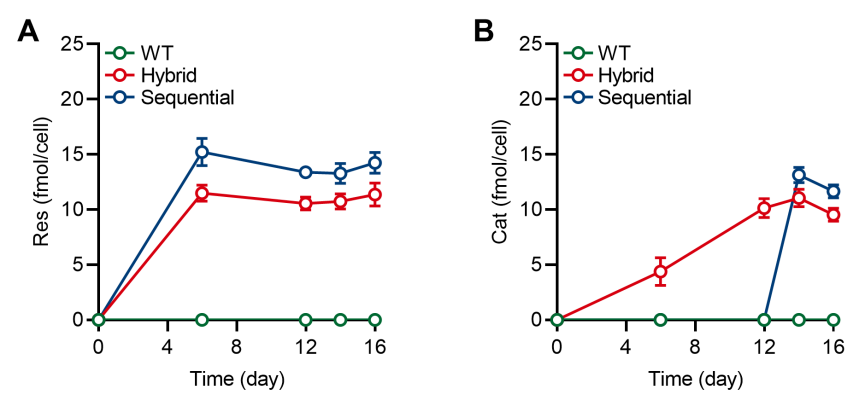


**Fig. S3.** The content of exogenous phytohormones in *H. pluvialis* cells treated with 200 μmol of Res and 100 μmol of Cat in hybrid and sequential feeding strategies. (A) The content of Res; (B) The content of Cat. Error bars in the figures represent the standard deviation of measurements from at least three samples.


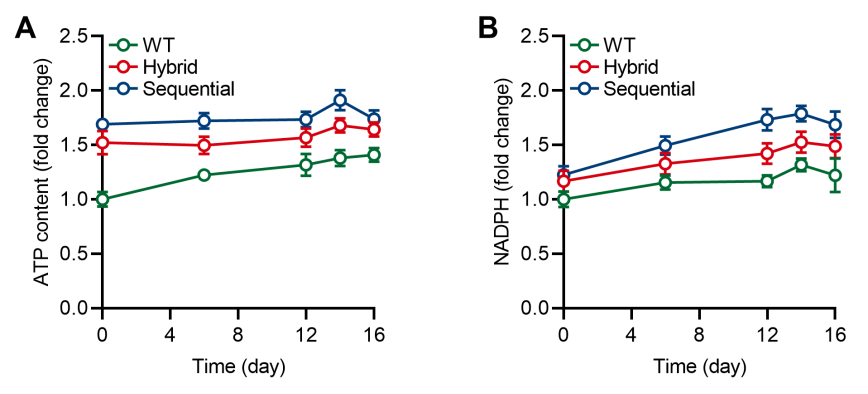


**Fig. S4.** The content of energy and reducing equivalent in *H. pluvialis* cells treated with 200 μmol of Res and 100 μmol of Cat in hybrid and sequential feeding strategies. (A) The content of ATP; (B) The content of NADPH. Error bars in the figures represent the standard deviation of measurements from at least three samples.


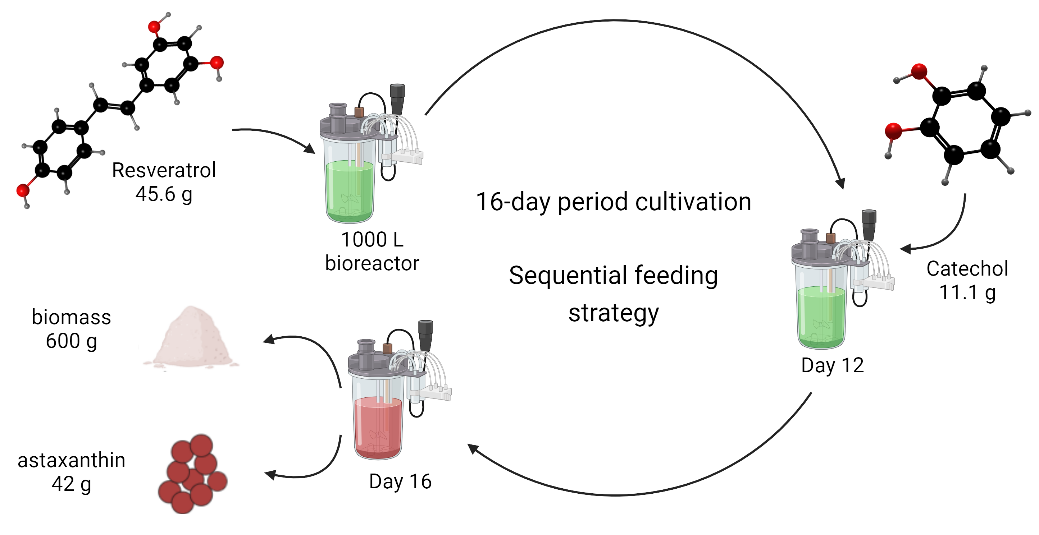


**Fig. S5.** Mass balance of *H. pluvialis* treated with 200 μmol of Res and 100 μmol of Cat in the sequential feeding strategy in a simulated 1,000-L scale based on the laboratory data from this study.
